# Using genetic comparisons of populations from Arizona, Mexico, and Texas to investigate fall armyworm migration in the American southwest

**DOI:** 10.1371/journal.pone.0289060

**Published:** 2023-11-27

**Authors:** Rodney N. Nagoshi, Ashley E. Tessnow, Yves Carrière, Jeff Bradshaw, Kyle Harrington, Gregory A. Sword, Robert L. Meagher

**Affiliations:** 1 Center for Medical, Agricultural and Veterinary Entomology, United States Department of Agriculture-Agricultural Research Service, Gainesville, Florida, United States of America; 2 Department of Entomology, Texas A&M University, College Station, Texas, United States of America; 3 Department of Entomology, University of Arizona, Tucson, Arizona, United States of America; 4 Department of Entomology, University of Nebraska-Lincoln, Lincoln, Nebraska, United States of America; Southeastern Louisiana University, UNITED STATES

## Abstract

Fall armyworm (FAW) is a global agricultural pest, causing substantial economic losses in corn and many other crops. Complicating efforts to control this pest is its capacity for long distance flights, which has been described in greatest detail for the central and eastern sections of the United States. FAW infestations are also routinely found in agricultural areas in southern Arizona, which lie beyond the western limits of the mapped migratory pathways. Climate suitability analysis found that the affected Arizona locations cannot support permanent FAW populations, indicating that these FAW most likely arise from annual migrations. A better understanding of this migration would provide insights into how large moth populations can move across desert habitats as well as the degree of gene flow occurring between FAW populations across the North American continent. In this study the Arizona populations were genetically characterized and compared to a selection of permanent and migratory FAW from multiple sites in the United States and Mexico. The results are consistent with migratory contributions from permanent populations in the states of Texas (United States) and Sinaloa (Mexico), while also providing evidence of significant barriers to gene flow between populations within Mexico. An unexpected finding was that two genetically distinct FAW subpopulations known as “host strains” have a differential distribution in the southwest that may indicate significant differences in their migration behavior in this region. These findings indicate that the combination of mitochondrial and *Z*-linked markers have advantages in comparing FAW populations that can complement and extend the findings from other methods.

## Introduction

The capacity of many agriculturally significant moth pests to migrate large populations over long distances has substantial ecological and economic consequences [1]. This behavior facilitates invasiveness both by increasing the likelihood of introduction into new locations and driving the rapid dissemination of the pest after establishment [2]. It can also greatly extend the geographical range of infestation well beyond the locations capable of supporting permanent populations. In this case the migratory behavior is recurring as the species is geographically limited during the less favorable part of the year but then expands its range as seasonal conditions improve [3].

An example of such a pest is the noctuid moth *Spodoptera frugiperda* (JE Smith) (Lepidoptera: Noctuidae), commonly known as fall armyworm (FAW), which is responsible for substantial economic losses in corn production and can cause significant yield losses in cotton, pasture/forage grasses, sorghum, millet, and alfalfa [4, 5]. FAW is a semi-tropical species that is not known to survive the prolonged freezing temperatures common to winters in most of North America [6–8]. Early viability studies estimated that permanent FAW populations are limited to regions south of 29°N latitude, which in North America approximates to Orlando, Florida and San Antonio, Texas [9–11]. A recent global climate suitability study placed this overwintering boundary further north at approximately 30°N latitude, which includes most of the Florida peninsula and the coastal regions of the Gulf coast [12]. With either boundary, the Gulf of Mexico acts as a barrier to limit the overwintering FAW populations from interacting at these latitudes, dividing the species into two distinct overwintering populations.

Despite this limited capacity to survive North American winters, the geographical range of FAW infestation consistently extends to southern Canada [7]. This indicates an annual migration of several thousand kilometers following pathways identified using genetic markers [13–16]. The populations overwintering in southern Texas and agricultural areas near the Mexico border are the source of the FAW infesting the central and most of the northeastern United States, while Florida populations are the primary contributors to FAW in the southeastern states of Georgia and South Carolina, as well as the eastern coastal states as far north as Maryland. Because FAW is primarily a nocturnal flier [17], this migration most likely occurs through a series of roughly six-hour continuous flights that can span multiple generations and necessitates locations along the migratory pathway that can support FAW during the day [18]. Field observations [7, 19] and modeling [18, 20] indicate that this sequential movement in the United States coincides with the northward progression of crop planting, which provides the abundance of plant hosts capable of supporting high density migration. Modeling and field studies indicate that this migratory behavior is also facilitated by and is probably dependent upon seasonal air transport systems that favor a northward and eastward movement of FAW into the central and eastern United States [18, 20–23].

Less is known about the origins of the FAW infestations in southern Arizona, which is outside the region where FAW migratory pathways were mapped. The Arizona climate is generally unfavorable for permanent FAW populations [12], with the major agricultural locations surrounded by desert and mountain habitats. These observations indicate that the infestations are most likely migratory in origin with the best candidates for source populations in southern Texas and Mexico based on proximity to Arizona and a better than marginal climate suitability.

To better define FAW populations and their migratory behavior in the southwestern United States, the Arizona FAW populations were characterized for genetic markers derived from the mitochondrial Cytochrome oxidase subunit I (*COI*) gene and the Z-chromosome-linked Triosephosphate isomerase (*Tpi*) gene. *COI* haplotypes show a differential distribution between FAW overwintering in Texas and Florida [24] that extends to the migratory populations originating from these sources [13, 25]. In addition, polymorphisms in *COI* can at least partially differentiate two populations denoted as host strains that differ in their distribution on different plant types in field surveys (reviewed in [26, 27]). The C-strain generally predominates in corn, sorghum, and cotton, while the R-strain can be found in all the hosts in North America (behaving as generalists in plant use) but appears to be more specifically associated with forage/pasture grasses, turf grasses, alfalfa, and millet in South American populations [28]. The two host strains are morphologically indistinguishable but are genetically distinct, differing in their *COI* haplotypes as well as in other strain defining molecular markers. However, the association of the molecular markers with host use is not absolute and therefore the strains as identified by markers are characterized as having a differential “preference” for different plant types.

There is evidence that the differences in host specificity are determined by genes on the *Z*-chromosome, which is supported by data showing that polymorphisms in *Tpi* exon sequences provide a more consistent and accurate determination of strain identity than *COI* [28–30]. In addition, the high genetic variability observed in an adjacent *Tpi* intron segment makes it a useful tool to compare populations, where it provided new insight into the biology of the host strains [28, 31]. Consequently, the *Tpi* gene is among the best tools to both identify strains and delineate how they might differ between geographical populations, supporting and complementing the well-studied *COI* markers.

In this study, the *COI* and *Tpi* markers in the Arizona FAW populations were compared to those from selected populations in the United States and Mexico. Insights obtained on the possible migratory origins of the Arizona populations and the overall migration pattern in North America are discussed. The further refinement of these genetic resources should facilitate their use in other parts of the world where FAW migratory behavior is not yet understood.

## Materials and methods

### Source and treatment of specimens

The FAW collected from Arizona and Nebraska were adult males obtained by pheromone trapping using plastic Universal moth traps (Unitraps) baited with a 2-component mix designated “Fall Armyworm-PSU” lure (Scentry Biologicals, Inc., Billings, Montana) and containing insecticide strips (Hercon Environmental Co., Emigsville, Pennsylvania). All other United States collections were from pheromone traps and described in previous studies (Table 1). The collections from Mexico include four colonies derived from populations in the states of Sinaloa, Durango, Tamaulipas, and Chiapas that had gone through <7 generations in culture at the time of their molecular analysis [15]. Field collections were made by sweep netting in Ciudad Mante and Ciudad Durango at approximately the same locations as the sources of the Tamaulipas and Durango colonies.

**Table 1:** Source information for collections.

**A. United States collections County, State**	**Year**	**Habitat**	**Type** ^**1**^	**Collector or Reference**
Pima, Arizona	2019	Corn	T	Y. Carriere
Pinal, Arizona	2019, 2021	Corn	T	Y. Carriere
Pinal, Arizona	2021	Alfalfa	T	K. Harrington
Maricopa, Arizona	2021	Alfalfa	T	K. Harrington
Yuma, Arizona	2021	Corn	T	J. Palumbo
Miami-Dade, Florida	2005	Corn	T	[24]
Orange, Florida	2012	Corn	T	[32]
Miami-Dade, Florida	2014	Corn	T	[32]
Manatee, Florida	2019	Corn	T	R. Meagher
Burke, Georgia	2021	Corn	T	R. Meagher
Clay, Nebraska	2011	Corn	T	[11]
Scotts Bluff, Nebraska	2012	Corn	T	J. Bradshaw
Scotts Bluff, Nebraska	2013	Corn	T	J. Bradshaw
Scotts Bluff, Nebraska	2014	Corn	T	J. Bradshaw
Scotts Bluff, Nebraska	2015	Corn	T	J. Bradshaw
Charleston, South Carolina	2011	Corn	T	[11]
Suffolk, Virginia	2011, 2013	Corn	T	[33]
Brazos, Texas	2004	Corn	T	[11]
Hidalgo, Texas	2007	Corn	T	[11]
Nueces, Texas	2011–12	Corn	T	[15]
Nueces, Texas	2015	Corn	T	R. Parker
Lubbock, Texas	2021	Corn	T	P. Porter
**B. Mexico Collections State**	**Year**	**Habitat**	**Type** ^**1**^	**Reference**
Chiapas	2013	Corn	C	[15]
Durango	2013	Corn	C	[15]
Sinaloa	2013	Corn	C	[15]
Tamaulipas	2013	Corn	C	[15]
Durango	2014	Corn	L	[15]
Tamaulipas	2014	Corn	L	[15]

^1^T = Trap; C = Colony; L = Larval collection.

Upon collection, the specimens were dried then mailed to CMAVE, Gainesville, Florida for DNA preparation. Nuclear and mitochondrial DNA were isolated from single specimens by homogenization in a 5-ml Dounce homogenizer (Thermo Fisher Scientific, Waltham, Massachusetts) in 1 ml of phosphate buffered saline (PBS, 20 mM sodium phosphate, 150 mM NaCl, pH 8.0). The homogenate was transferred to a 2-ml microcentrifuge tube and pelleted by centrifugation at 6000 g for 5 minutes at room temperature. The pellet was resuspended in 400 μl Genomic Lysis buffer (Zymo Research, Orange, California) by vortexing and incubated at 55°C in a dry bead bath for at least one hour. Debris was removed by centrifugation at 10,000 rpm for 5 minutes. The supernatant was transferred to a Zymo-Spin III column (Zymo Research, Orange, California) and processed according to manufacturer’s instructions but with elution using 100 μl of either distilled water or TE.

Additional moth samples were collected in 2021 from Yuma and Pinal counties in Arizona, Lubbock county, Texas and Burke county, Georgia for use in the SNP C analysis. These were preserved in >70% ethanol and stored at -20°C. DNA was extracted from these samples using a Gentra Puregene Tissue Kit (Qiagen, Germantown, Maryland, USA) in accordance with the manufacturer’s protocol.

### DNA sequence analysis

The relevant segments from the *COI* and *Tpi* genes were amplified by polymerase chain reaction (PCR) amplification in a 30-μl reaction mix containing 3 μl of 10X manufacturer’s reaction buffer, 1 μl 10mM dNTP, 0.5 μl 20-μM primer mix, 1 μl DNA template (between 0.05–0.5 μg), 0.5 units Taq DNA polymerase (New England Biolabs, Beverly, Massachusetts) with the remaining volume water as describe in [34]. The thermocycling program was 94°C (1 min), followed by 33 cycles of 92°C (30 s), 56°C (45 s), 72°C (45 s), and a final segment of 72°C for 3 min. Primers used for the PCR amplification of COIB are c891F (5’-TACACGAGCATATTTTACATC-3’) and c1472R (5’-GCTGGTGGTAAATTTTGATATC-3’). Primers for the *Tpi* segment used for strain identification and that includes the TpiI4a200 intron fragment are t412F (5’-CCGGACTGAAGGTTATCGCTTG-3’) and t1140R (5’-GCGGAAGCATTCGCTGACAACC-3’). All primers were synthesized by Integrated DNA Technologies (Coralville, Iowa).

The PCR products were separated by agarose gel electrophoresis and purified using the Zymoclean Gel DNA Recovery Kit (Zymo Research, Orange, California). The isolated fragments were directly analyzed by DNA sequencing performed by either Azenta Life Sciences (Chelmsford, Massachusetts) or Eurofins Genomics (Louisville, Kentucky).

#### Identification of the COIB CSh[1–4] haplotype categories

**Fig 1:**
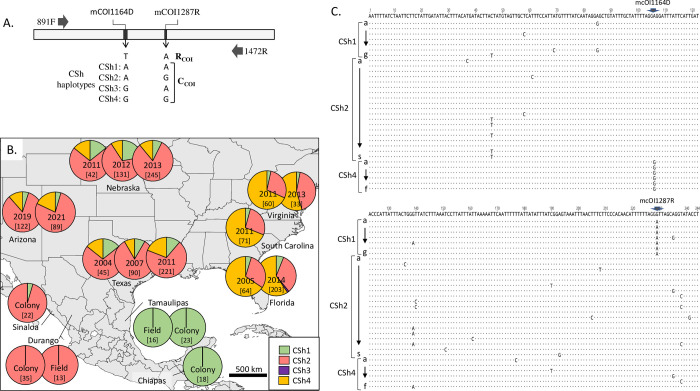
Description of the COIB haplotypes and their distribution. A: Molecular map of the COIB segment. Block horizontal arrows indicate primer locations for PCR and DNA sequencing. The relative locations of the mCOI1164D and mCOI1287R polymorphisms are indicated with the empirically observed nucleotides listed below. The T_1164_A_1287_ combination identifies the R-strain based on *COI* (R_COIB_) while the other combinations define the COIB-based C-strain haplotypes that are designated CSh1-CSh4. B: Distribution of COIB haplotypes at different locations and times. Numbers in brackets indicate samples tested. C: C-strain COIB variants (C_COIB_) from the TX collections. Each CSh haplotype is based on mCOI1164D and mCOI1287R, but polymorphisms at other sites also occur. The top line is the consensus sequence with dots below indicating agreement with the consensus. The locations of mCOI1164D and mCOI1287R are indicated.

The relevant *COI* haplotypes for this study are from the 3’ half of the gene (COIB) and are defined by two polymorphic sites designated mCOI1164D and mCOI1287R (Fig 1A). The configuration T_1164_A_1287_ (the R_COIB_ strain marker) predominates in FAW found in R-strain preferred host plants [29, 30]. Four variants define C_COI_, A_1164_A_1287_ (CSh1), A_1164_G_1287_ (CSh2), G_1164_A_1287_ (Csh3), G_1164_G_1287_ (CSh4) and are associated with the use of C-strain hosts (collectively designated as the C_COIB_ markers). The other possible pairwise combinations have yet to be observed. The CSh3 haplotype is rare while CSh[1,2,4] are typically found in all locations. However, CSh2 and CSh4 show reproducible differences in frequency depending on location, with the initial observation being that CSh2 dominates in Texas while CSh4 is the majority in Florida [13, 24]. The COIB analysis were from previous studies for all collections except for those from Arizona and Nebraska (2012–2013) [13, 14, 33].

#### Identification of strains using *Tpi* and characterization of TpiI4a200

Versions of the full length FAW fourth exon and following intron (TpiI4) for the C-strain and R-strain are included in GenBank sequences KT336236 and KT336231, respectively. The *Tpi* fourth exon contains three polymorphic sites (gTpi165Y, gTpi168Y, and gTpi183Y) that exhibit biases based on associations with different plant hosts [29]. The gTpi183Y polymorphism showed the most consistent correspondence with the appropriate plant host and so was used as the diagnostic marker for *Tpi*-based strain identity, with C_183_ defining the C-strain (C_Tpi_) and T_183_ the R-strain (R_Tpi_). Out of 160 C_183_ sequences only four showed a disagreement in strain identity with gTpi165Y or gTpi168Y. One complication with using the *Z*-linked *Tpi* gene as a marker is that male FAW have two *Z*-chromosomes and so can be heterozygous for different *Tpi* alleles. Because we directly sequence the PCR product amplified from the nuclear genome, heterozygosity for polymorphisms within the region of interest will produce ambiguous sequence data in the form of overlapping chromatogram curves. These were not included in the sequence analysis of TpiI4a200 with one exception. A subset of the heterozygotes are C_Tpi_/R_Tpi_ hybrids produced by mating between strains. These were identified by a C/T overlap at site gTpi183Y in the sequence chromatogram and were designated H_Tpi_ in the strain analysis.

The intron immediately adjacent to this exon (TpiI4, [31]) displays high sequence variation and is of variable size because of frequent indels. This high variability results in many heterozygous specimens displaying ambiguous TpiI4 sequences due to overlapping polymorphisms. To increase the number of useable sequences, the analysis was limited to the first 60% of the intron that approximated 200-bp (size uncertainty due to indels). This segment is designated TpiI4a200 and was isolated and sequenced using the same set of *Tpi* primers.

#### Identification of strains using *Z*-chromosome markers

Samples collected in 2021 were genotyped using the SNP C TaqMan Real-Time diagnostic assay described in [35]. In brief, real-time PCR assays were conducted as 10 μl reactions containing 1 μl of DNA, 5 μl of TaqMan Genotyping Master Mix (Applied Biosystems, Waltman, Massachusetts), and 0.5 μl of 40×Custom ThermoFisher TaqMan assay containing the primers and hydrolysis probes (sequences included in S1 Table). PCR began at 95°C for 10 min, followed by 40 cycles oscillating between 95°C for 15 s and 60°C for 1 min. Fluorescence was recorded between each oscillation. Alleles were called using QuantStudios Real-Time PCR Software v1.7.1 (Applied Biosystems).

### Analysis and presentation of data

DNA sequence analysis including alignments and Neighbor-Joining [36] comparison of sequences were performed using Geneious Prime 2021.1.1 (Biomatters, Auckland, New Zealand). The generation of graphs were done using Excel and PowerPoint (Microsoft, Redmond, WA). Sequences used in the phylogenetic analysis have been deposited into GenBank. All DNA haplotypes described are available in the NCBI Genbank database as follows: TpiI4a200 variants (accession numbers OQ732378-OQ732385), COIB variants (accession numbers OQ725031-62). All maps not attributed to other sources were created using the Free and Open Source QGIS (https://www.qgis.org/en/site/).

## Results

### COIB CSh-haplotype profiles

Frequency differences between the four COIB C-strain h-haplotypes (CSh1-4) can reliably distinguish FAW that winter in southern Florida from those in southern Texas as well as the migratory populations that derive from these sources. Specifically, the CSh2 haplotype is the majority form in Texas while CSh4 predominates in Florida. The CSh1 haplotype is a minority form in both locations with generally higher frequencies in Texas, while CSh3 is rarely observed in all collections. The data from multiple studies are summarized in Fig 1B for Texas, Florida, South Carolina, Virginia, and Mexico, and combined with new COIB analysis from Nebraska and Arizona. The results describe the consistency of the CSh haplotype profiles in collections at or near the overwintering locations in Texas and Florida over multiple years, as well as the reproducibility of the profiles at the migratory destinations as exemplified for migration from Texas to Nebraska and from Florida to South Carolina and Virginia. The persistence of the CSh frequency profiles between the source and destination populations even after a migration of 2000 kilometers suggests the movement of large populations with no bottlenecks.

The CSh profiles from colonies and field collection in Mexico differed from those observed in TX and FL [15]. Colonies derived from Sinaloa and Durango populations were almost entirely CSh2, with only a small number of the CSh1 category found in Sinaloa (Fig 1B). In contrast, the colonies from Tamaulipas and Chiapas were entirely CSh1. Field collections from Durango and Tamaulipas gave results identical to the colonies [15].

Collections from Arizona in 2019 and 2021 contained the CSh1, CSh2, and CSh4 haplotype categories at frequencies that resemble those found in Texas and Nebraska (Fig 1B). The Arizona profile is very different from that characteristic of populations from Florida or previously described for the Mexican states of Tamaulipas and Chiapas. In contrast, the Arizona data resemble that of Sinaloa and Durango in that all locations are dominated by the CSh2 haplotype.

**Fig 2:**
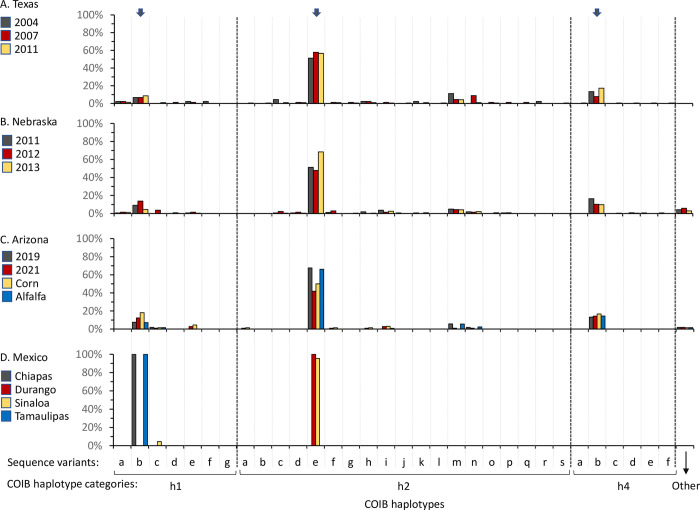
Bar graphs showing the frequencies of the COIB variants. Data are shown for Texas, Nebraska, Arizona, and the four states examined in Mexico. All variants had at least one specimen in the Texas collections. Vertical arrows at top of graph show the predominant variant for each CSh haplotype. The “other” category represents variants not found in the surveyed Texas collections.

A more detailed analysis of haplotype composition was based on a survey of 377 specimens from Texas collections made in 2004 (n = 45), 2007 (90), and 2011 (221), which identified additional sequence polymorphisms that produced 30 variants, with six found for CSh1 (CSh1[a-g]), 19 for CSh2 (a-s), and five for CSh4 (a-f) (Fig 1C). Despite this genetic variability, each h-haplotype category had a predominant variant with CSh1[b] making up 70% of the CSh1 specimens, CSh2[e] at 77% of CSh2, and CSh4[b] predominating (91%) its category (Fig 2A). This pattern is also observed in the NE collections (CSh1[b] = 74%, CSh2[e] = 81%, CSh4[b] = 71%), and in Arizona (CSh1[b] = 78%, CSh2[e] = 88%, CSh4[b] = 88%) (Fig 2B and 2C). Although the Mexico collections differed in their h-haplotype profiles, they shared the same biases as the CSh1[b] haplotype was the only one observed in Chiapas and Tamaulipas, while CSh2[e] predominated in Sinaloa and Durango (Fig 2D).

### The distribution of the TpiI4 intron segment variants

**Fig 3:**
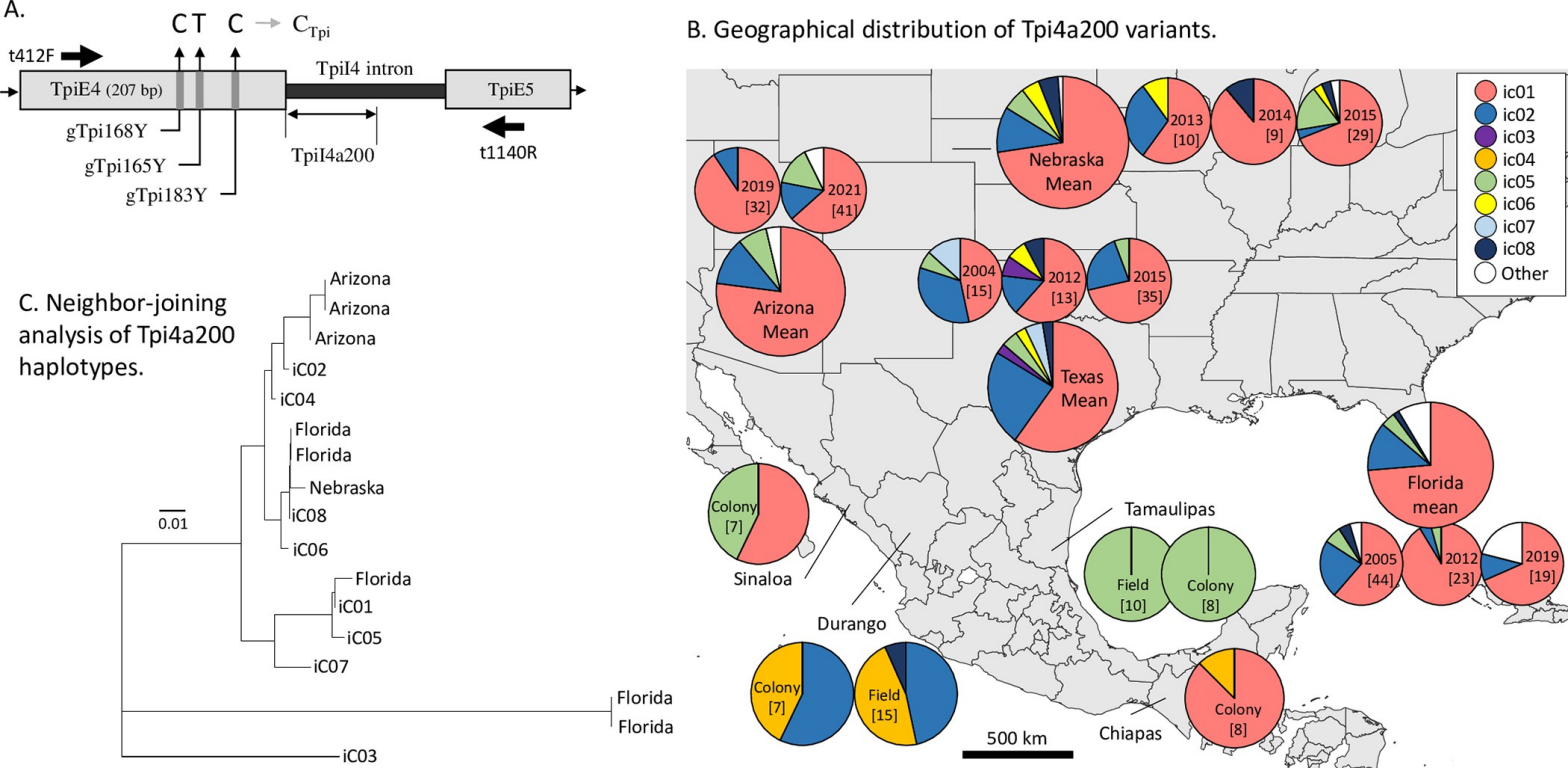
Description of the TpiI4a200 segment and the distribution of TpiI4a200 variants. A: Molecular map of the relevant portion of the *Tpi* gene showing two exon and the TpiI4 intron. Large arrows show location of primers used for PCR and DNA sequencing. The TpiE4 exon has three sites that show strain-biased polymorphisms with gTpi183Y showing the most consistent correspondence with host plant. B: Distribution of TpiI4a200 variants at different locations and times from same or similar locations as described for COIB with the Mexico and Arizona collections the same as used for COIB. The “other” category represents variants not found in the surveyed Texas collections. The number of specimens with unambiguous haplotypes is in brackets for each collection. C: Neighbor-Joining comparisons of the Texas TpiI4a200 haplotypes along with the unique sequences observed in Florida, Nebraska, and Arizona.

TpiI4a200 is an approximately 200-bp segment from a highly variable intron of the *Tpi* gene (Fig 3A, [28]). Of the approximately 250 specimens sequenced from Texas and Mexico for TpiI4a200, most gave ambiguous data because of heterozygosity to polymorphic sites and indels. A total of 118 unambiguous TpiI4a200 sequences were obtained from which eight haplotypes (iC01-8) were identified. The iC01 variant was the most frequent observed in Texas, making up on average about 60% of the collections (Fig 3B). The frequencies of the remaining haplotypes were highly variable from year to year, with iC02 the next most frequent in Texas at about 20%. The Florida collections shared variants iC01, iC02, iC05 and iC08 with Texas and contained six specimens with four haplotypes unique (other, Fig 3) to Florida. Of the 48 TpiI4a200 specimens analyzed in the Nebraska collections, only one carried a haplotype that was not represented in Texas.

The Arizona collections resembled those from Texas, Florida, and Nebraska in that they displayed a majority iC01 composition in both 2019 (91%) and 2021 (62%), with iC02 a significant contributor. The iC05 variant was also present in 2021 (17%) along with two haplotypes (three specimens) not found in the Texas collections. These differ from the analogous unique haplotypes observed in Nebraska and Florida (Fig 3C).

Once again, the collections from Mexico were most divergent in their frequency profiles with each location differing substantially from the others. Both the colony and field collection from Tamaulipas was comprised of a single haplotype, iC05, that made up on average only 4% of the Texas collections but was a substantial component of the Sinaloa colony at 43% (Fig 3B). The rest of the Sinaloa colony was iCO1, which was also the majority form in the Chiapas colony. A second haplotype, iC04, was found in Chiapas as well as in both the colony and field collections from Durango. This haplotype appears to be rare or perhaps even absent in the United States as it was not detected in the Texas, Florida, or Nebraska collections.

### The distribution of host strains

**Fig 4:**
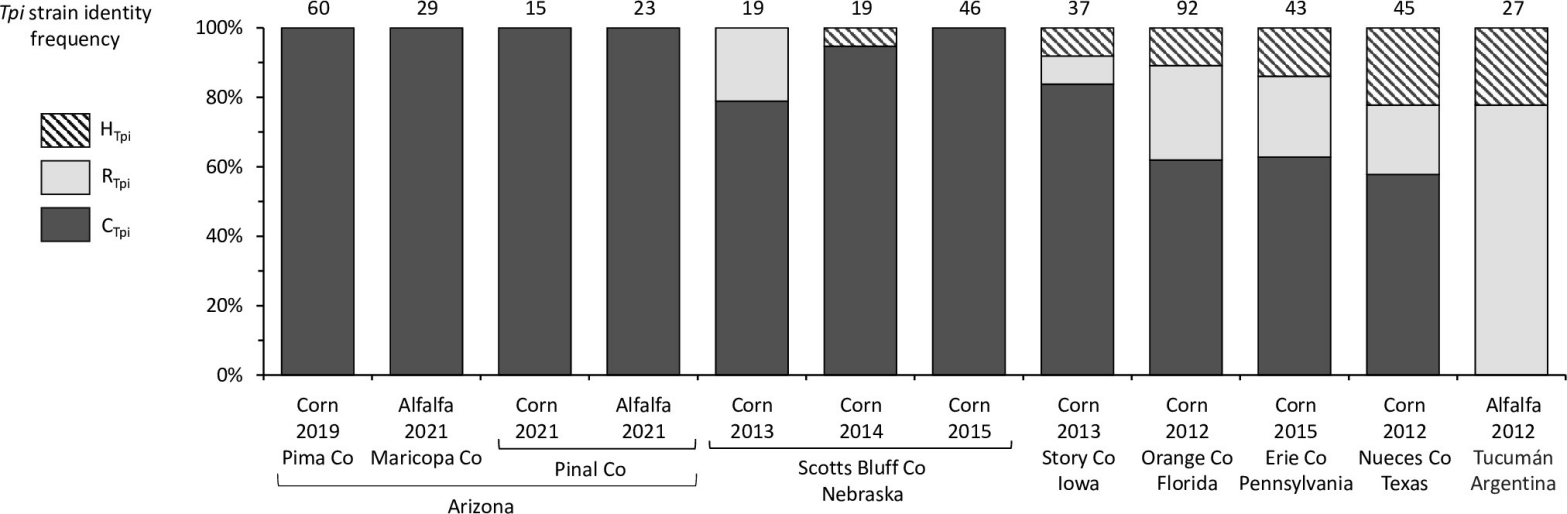
Bar graphs describing the frequencies of the strain types as categorized by the gTpi183Y marker. The specimens from Iowa, Pennsylvania, and Argentina are from earlier studies [27, 40]. Numbers above columns indicate samples analyzed.

The FAW host strains (C and R) are most commonly identified using either the *COI* (C_COIB_, R_COIB_) or *Tpi* (C_Tpi_, R_Tpi_) markers. These are generally in agreement, with the *Tpi* marker considered more accurate because of its more consistent association with strain-specific host plants [29, 30]. Disagreements between *COI* and *Tpi* do occur and are likely due to the lesser strain-specificity of the *COI* polymorphisms as well as cross-hybridization between strains that can dissociate the mitochondrial from the *Z*-chromosome markers [27, 35]. While collections from corn generally have C_COIB_ or C_Tpi_ majority, there is usually a R_COIB_ or R_Tpi_ minority with evidence of seasonal variation in the relative frequencies [37, 38]. This is particularly true for pheromone trap collections in cornfields, a method that samples for adult male specimens in the vicinity of corn but that may not have developed on corn. The frequency of R-strain markers in collections from corn is indicated by surveys in Nebraska, Florida, Iowa, Pennsylvania, and Texas (Fig 4). As a comparison, the *COI* and *Tpi* haplotypes found in a larval collection from alfalfa in Argentina is dominated by the R-strain polymorphisms, indicative of this host being preferred by the R-strain [39, 40]. Additional Z-chromosome SNP markers also differ between strains and provide similar results to *Tpi* strain identifications [35].

**Fig 5:**
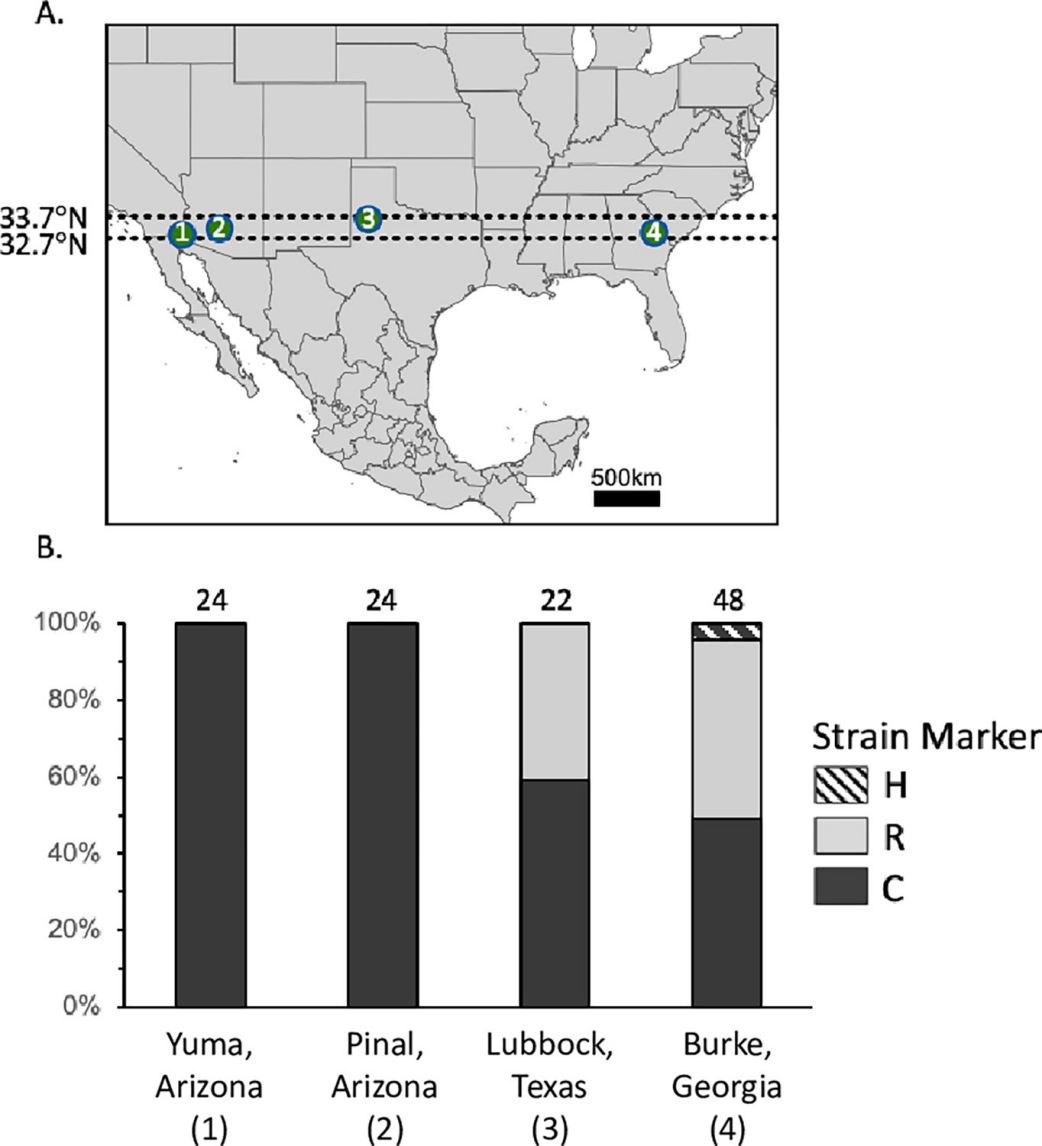
Strain composition after sampling across a single latitude in 2021. A: Sampling map for fall armyworm moths collected from September 7- October 26, 2021. All captures occurred within a single degree of latitude (32.7–33.7°N). Map location 1–4 represent Yuma, Arizona, Pinal, Arizona, Lubbock, Texas, and Burke, Georgia, respectively. B: Bar graph describing the frequencies of each strain as categorized by the SNP C Z-chromosome marker. The number above each column indicates the number of samples analyzed.

Notably, the R-strain appears to be rarely found in AZ. Only two R_COIB_ specimens were found out of 127 total specimens tested and no R_Tpi_ were detected in a total of 133 specimens. No evidence of the R-strain using either marker was found in pheromone trap surveys in alfalfa, which contrasts with the results from larval alfalfa collections in Argentina (Fig 4). A similar finding was observed in the surveys of FAW in Mexico, although these collections were limited to corn habitats. Neither R_COIB_ or R_Tpi_ were found in the four colonies or two field collections. When using *Z*-chromosome markers to compare strain proportions of moths collected across four locations at the same latitude (32.7–33.7°N) in the same month (September-October 2021), there was again no evidence of R-strain occurring in Arizona, while both strains were evident in nearly even proportions in Texas and Georgia (Fig 5).

## Discussion

The mitochondrial *COI* and *Z*-linked *Tpi* genes have been extensively used in FAW studies and thereby provide a comprehensive sequence database that can be applied to genetically compare geographical populations. A previous study found evidence using COIB markers that FAW populations in Mexico are highly stratified, with FAW from the western states of Sinaloa and Durango differing in haplotype profile from the eastern state of Tamaulipas and the southern state of Chiapas (Fig 1B, [15]). Our reanalysis of the same collections using TpiI4a200 confirmed and extended the evidence of stratification, with all four states showing significant differences in haplotype composition (Fig 3B). The Mexico findings should be considered preliminary since much of the data come from colonies where haplotype frequencies can change due to inbreeding and potential bottlenecks. This could, for example, explain why the number of haplotypes found in the Mexico collections is low relative to other overwintering areas such as Texas. However, field collections made the following year in Tamaulipas and Durango were consistent with the colony results, suggesting that the colonies approximated the haplotype profile of the source populations in these states. Still, data are limited to a two-year collection window and the consistency of the haplotype ratios in Mexico over time remains to be determined.

**Fig 6:**
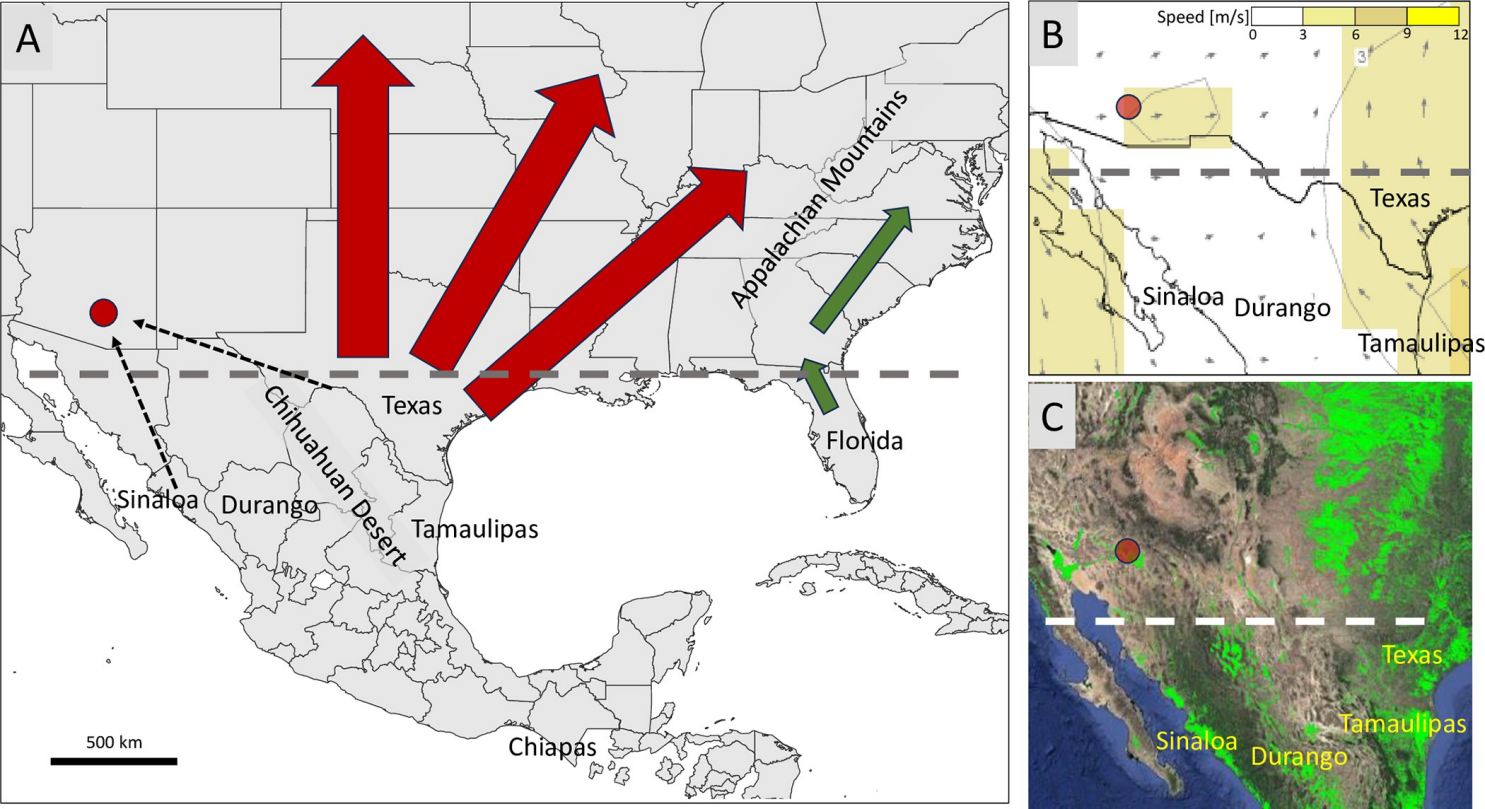
Maps related to FAW migration, agricultural activity, and seasonal wind systems. Red oval approximates location of primary agricultural areas in southern Arizona. Horizontal dashed line approximates overwintering boundary. A: Map showing known FAW migration pathways from Texas (block arrows). Putative migration into Arizona indicated by red dashed lines. B: Air transport patterns in April at 925 mb (approximately 762 m AGL); graphics provided by the International Research Institute for Climate and Society, Columbia University, https://iri.columbia.edu [42, 43]. April approximates the beginning of the FAW migration season [7, 18]. C: Map of croplands showing agricultural activity (green). Image is courtesy of U.S. Geological Survey at https://www.usgs.gov/apps/croplands/app/map.

A surprising observation was the significant differences in both the COIB and TpiI4a200 haplotypes from Tamaulipas compared to those from Texas (Fig 3B). The collection site in Mante, Tamaulipas is about 400 km flying distance from agricultural regions along the southern Texas border, which should be within the migratory range of FAW (Fig 6A). The CSh2 and iC05 haplotypes that make up the entirety of the Tamaulipas collections are found in Texas at mean frequencies of 11% and 4% respectively, indicating that if exchanges between these populations occur, they are not of sufficient magnitude to substantially alter the haplotype profiles. The same must also be true for the FAW in the surveyed Mexican states if the stratification observed between the collections is representative of field populations. The reasons for such apparent limitations in migration in Mexico are uncertain but are likely influenced by seasonal wind vectors that appear to be generally inconsistent in orientation and weak in magnitude (S1 Fig and Fig 6B), combined with the physical barriers of desert and mountain habitats that separate the major agricultural locations (Fig 6C). Mexico has been identified as a complex transition zone comprised of several major biogeographic regions with multiple potential boundaries to historical gene flow [41]. One example of the latter is the Chihuahuan Desert that extends through central Mexico that could challenge FAW viability through temperature extremes, dehydration, and inadequate food supply. The nocturnal flight behavior of FAW with the presumed need for daytime rest areas increases the plausibility of such barriers, particularly if prevailing winds are weak.

The consistent observation of FAW infesting the cornfields of southern Arizona means that either permanent FAW populations are possible in the region contrary to the predictions of climate suitability analyses or the migrations into Arizona routinely occur despite the challenging terrain, sparse agriculture, and unfavorable seasonal wind patterns. With respect to migration, of the possible sites capable of sustaining a permanent population described in this study, Florida and Chiapas are too far away, Tamaulipas is unlikely given the evidence of its segregation from FAW in neighboring Texas, and the Durango TpiI4a200 profile is substantially different from that observed in Arizona. In contrast, the haplotype composition of the Sinaloa and Texas collections are compatible with either one or both contributing to the FAW infestations in Arizona (Fig 6A). FAW populations from southern California have not been assessed to date. Although these cannot be ruled out as a potential source of migrants for Arizona populations, they are also not considered climatically suitable locations for permanent populations and may themselves be the result of colonization by migrants from the same sources [12].

An unexpected finding was the absence of the R-strain in Arizona (Figs 4 and 5). The R-strain is frequently found in host plants normally associated with the C-strain, which had been attributed to either plasticity in the behavior or incomplete specificity of the available strain markers. An alternative explanation that we favor is that the R-strain in North America is behaving as a generalist with respect to the selection of host plants, with host specificity limited to the C-strain, while the R-strain in South America is more consistently host specific [28]. The absence of the R-strain even in habitats dominated by R-strain-preferred alfalfa suggests the possibility of strain differences in migration. It is possible that the desert and mountain terrain surrounding the collection sites in Arizona are more restrictive to the R-strain than the C-strain. The R-strain was also shown to be absent in Ecuador [44], but Arizona would be the first example of strain exclusivity at a location where infestations are dependent on annual migration.

The scenario described here for Arizona where the necessity of long-distance migration is indicated by climate suitability projections is relatively unusual outside of North America. In most of South America the FAW infestation range generally overlaps habitats that can support permanent populations [12], obviating any need to assume migration. One notable exception is in southern Argentina where climate suitability analysis indicates that infestations there are probably of migratory origin. Genetic studies based on whole genome comparisons generally show little evidence of genetic differentiation in the region, preventing the identification of migratory source populations by this approach [45, 46]. There is recent evidence of genetic structure in Argentina, which may be evidence of an endogenous population, but it has not been demonstrated that the observed pattern is reproducible over time [47].

Although a native to the Americas, FAW has recently become a global concern with its discovery in western Africa in 2016 [48] that was followed by a remarkably rapid spread to much of Asia and Australia by 2020 [49, 50]. This movement was presumably facilitated by the migratory ability of FAW though specific pathways have yet to be delineated. It has been proposed based on the temporal pattern of FAW captures and migration modeling using wind transport systems that annual migrations analogous to that described in North America are occurring in southeastern Asia. Proposed pathways include migration from Myanmar into the Yangtze River Delta, then into Northern China, South Korea, and finally Japan [50–53]. While the data are suggestive that a northward migration is occurring, the identity of the source populations and the details of the migratory pathways remain speculative.

In summary, these results demonstrate that the combination of COIB and TpiI4a200 markers can produce important insights on FAW population structure and behavior not easily attained by other approaches. COIB polymorphisms are particularly sensitive to identifying partially isolated populations that still exhibit significant gene flow. This is because COIB is maternally inherited and so is solely dependent on the movement of females; males make no contribution to the haplotype profile of a population. Meanwhile, the *Z*-linked and highly polymorphic TpiI4a200 segment has the dual function of facilitating the genetic analysis of the host strains, while providing genetic variation that is contributed by both sexes that can be used to differentiate populations. The markers have been instrumental in describing FAW population in North America and in this study provide evidence of genetic structure in Mexico that suggests significant barriers to migration while also supporting Texas and Sinaloa FAW as plausible sources of infestations in southern Arizona. The methodology has the potential to be similarly productive if applied to other locations where the migratory pathways and strain distributions are less defined.

## Supporting information

S1 Table Primer and probes sequences used to amplify diagnostic SNP C on the Z-chromosome of fall armyworm. The FAM probe is specific to the R-strain while the VIC probe is specific to the C-strain.
(DOCX)

S1 Fig Air transport patterns at 925 mb (approximately 762 m AGL) for all months of the year. Graphics provided by the International Research Institute for Climate and Society, Columbia University, https://iri.columbia.edu.
(TIF)
