# Supplementary material for: Using genetic comparisons of populations from Arizona, Mexico, and Texas to investigate fall armyworm migration in the American southwest
Source: PLoS One. 2023 Nov 27;18(11):e0289060. doi: 10.1371/journal.pone.0289060 (PMC10681194; doi:10.1371/journal.pone.0289060)
Supplement: S1 Table — The FAM probe is specific to the R-strain while the VIC probe is specific to the C-strain. (DOCX) [file pone.0289060.s001.docx]

Supplementary Table S1. Primer and probes sequences used to amplify diagnostic SNP C on the Z-chromosome of fall armyworm. The FAM probe is specific to the R-strain while the VIC probe is specific to the C-strain.

| Forward Primer | 5'-TGACAGCATTGATGTGCTGGAT |
| --- | --- |
| Reverse Primer | 5'-CGCCGGAGCGTTACAGA |
| FAM Probe Sequence | 5'-FAM-CGCTACCAAAGCCAG-MGB-NFQ |
| VIC Probe Sequence | 5'-VIC-CGCTACCAGAGCCAG-MGB-NFQ |
