# Supplementary figures and images for: Using genetic comparisons of populations from Arizona, Mexico, and Texas to investigate fall armyworm migration in the American southwest
Source: PLoS One. 2023 Nov 27;18(11):e0289060. doi: 10.1371/journal.pone.0289060 (PMC10681194; doi:10.1371/journal.pone.0289060)

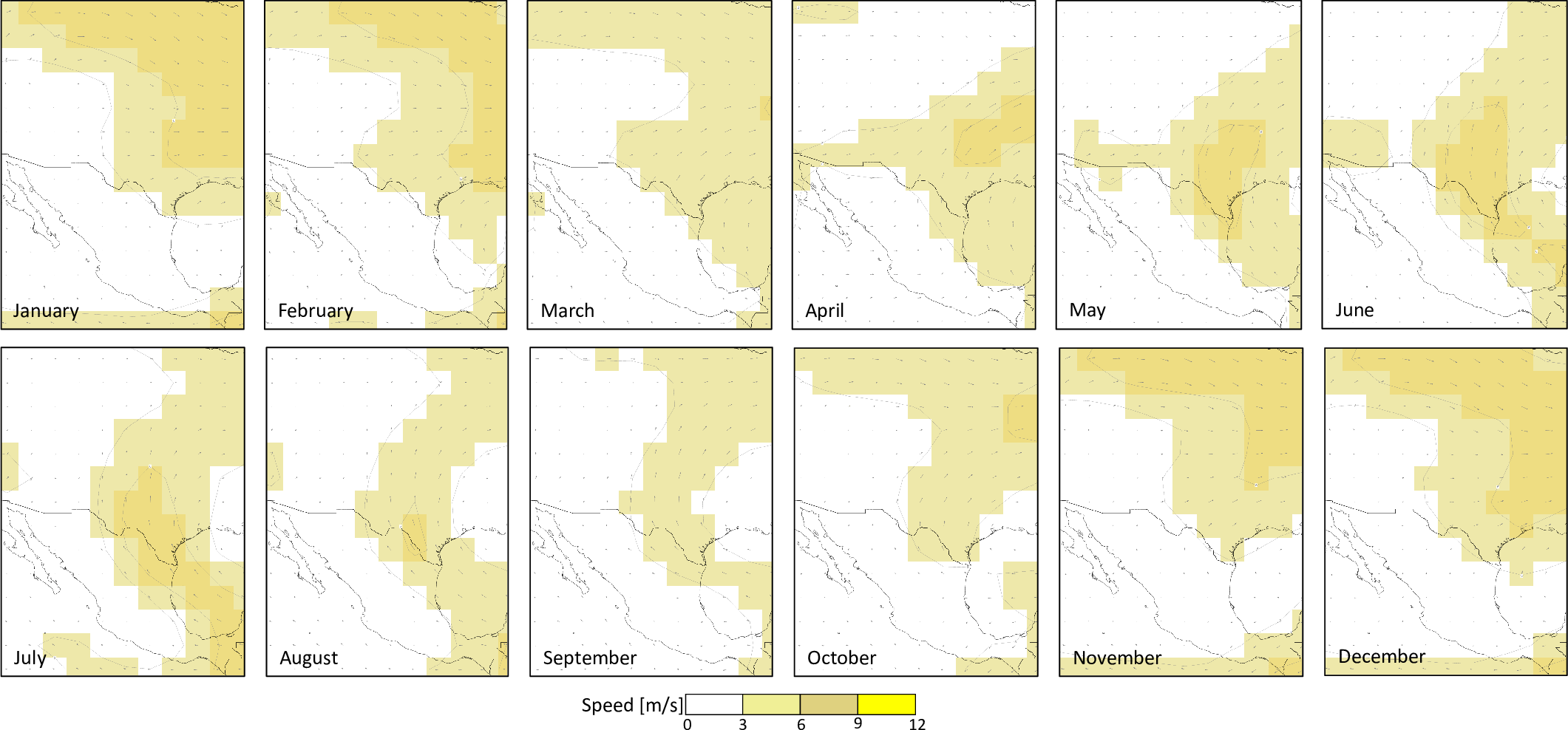

Supplement: S1 Fig — Graphics provided by the International Research Institute for Climate and Society, Columbia University, https://iri.columbia.edu. (TIF) [file pone.0289060.s002.tif]
